# Expression and function of human hemokinin-1 in human and guinea pig airways

**DOI:** 10.1186/1465-9921-11-139

**Published:** 2010-10-07

**Authors:** Stanislas Grassin-Delyle, Emmanuel Naline, Amparo Buenestado, Paul-André Risse, Edouard Sage, Charles Advenier, Philippe Devillier

**Affiliations:** 1 Laboratory of pulmonary pharmacology UPRES EA220, Foch Hospital, University Versailles-Saint Quentin en Yvelines, 11 rue Guillaume Lenoir, 92150 Suresnes, France; 2 Meakins-Christie Laboratories, Department of Medicine, McGill University, Montreal, QC, Canada; 3 Department of thoracic surgery, Foch Hospital, University Versailles-Saint Quentin en Yvelines, 40 rue worth, 92150 Suresnes, France

## Abstract

**Background:**

Human hemokinin-1 (hHK-1) and endokinins are peptides of the tachykinin family encoded by the *TAC4 *gene. *TAC4 *and hHK-1 expression as well as effects of hHK-1 in the lung and airways remain however unknown and were explored in this study.

**Methods:**

RT-PCR analysis was performed on human bronchi to assess expression of tachykinin and tachykinin receptors genes. Enzyme immunoassay was used to quantify hHK-1, and effects of hHK-1 and endokinins on contraction of human and guinea pig airways were then evaluated, as well as the role of hHK-1 on cytokines production by human lung parenchyma or bronchi explants and by lung macrophages.

**Results:**

In human bronchi, expression of the genes that encode for hHK-1, tachykinin NK_1_-and NK_2_-receptors was demonstrated. hHK-1 protein was found in supernatants from explants of human bronchi, lung parenchyma and lung macrophages. Exogenous hHK-1 caused a contractile response in human bronchi mainly through the activation of NK_2_-receptors, which blockade unmasked a NK_1_-receptor involvement, subject to a rapid desensitization. In the guinea pig trachea, hHK-1 caused a concentration-dependant contraction mainly mediated through the activation of NK_1_-receptors. Endokinin A/B exerted similar effects to hHK-1 on both human bronchi and guinea pig trachea, whereas endokinins C and D were inactive. hHK-1 had no impact on the production of cytokines by explants of human bronchi or lung parenchyma, or by human lung macrophages.

**Conclusions:**

We demonstrate endogenous expression of *TAC4 *in human bronchi, the encoded peptide hHK-1 being expressed and involved in contraction of human and guinea pig airways.

## Background

The mammalian tachykinins are a family of structurally related peptides which are derived from three distinct genes. *TAC1 *encodes for substance P (SP) and neurokinin A (NKA) through alternative splicing, while *TAC3 *encodes for neurokinin B (NKB) [1,2]. *TAC4 *was identified recently in lymphoid B haematopoietic cells of the mouse bone marrow and encodes for hemokinin-1 (HK-1) [3]. The same peptide is encoded by the rat *TAC4 *[4] and is consequently named rat/mouse hemokinin-1 (r/mHK-1). In human, *TAC4 *encodes for hemokinin-1 (hHK-1), but its sequence is different from its murine counterpart. A more detailed analysis of the *TAC4 *gene in humans showed that it is spliced into four alternative transcripts (α, ß, γ and δ) that give rise to four different peptides which have been named endokinins, endokinin A (EKA), B (EKB), C (EKC) and D (EKD). Extensive *TAC4 *expression has been shown in a number of murine tissues including brain, spleen, stomach, skin, breast, bone marrow, thymus, prostate, uterus, skeletal muscle, lymph node, eyes, as well as in lung [5]. In human, *TAC4 *expression has been observed in several tissues including brain, cerebellum, thymus, prostate, testis, uterus, adrenal gland, fetal liver and spleen for *αTAC4*; heart, liver adrenal gland, bone marrow, prostate and testis for *βTAC4*, whereas *γ-*and *δTAC4 *where ubiquitously expressed, with the most prolific expression in the adrenal gland and placenta [4-6]. *TAC4 *expression in human lung was reported in multi-tissue cDNA expression panels, but without distinction of the different anatomical entities (bronchi, parenchyma...) [4,6].

The biological action of tachykinins are mediated by at least three different transmembrane G-protein coupled receptors, namely NK_1_, NK_2 _and NK_3 _receptors which are stimulated preferentially, but not exclusively by SP, NKA and NKB, respectively [7-9]. r/mHK-1 has similar affinity to SP at the human NK_1 _receptor [4,6,10-12], while hHK-1 binds to the human NK_1 _receptor with a 14-fold lower affinity than SP [4]. Human HK-1 also binds to the human NK_2 _and NK_3 _receptors, with an affinity about 200-250-fold lower than for NK_1 _receptors [4,10,13].

HK-1 is involved in a variety of biological effects. Many studies have focused on its actions on immunological regulation and inflammation. Indeed, r/mHK-1 was initially found to be an important growth and survival factor for mouse early B-cells [3,14-16] and can play a role in murine T-cell development [15]. With respect to smooth muscle preparations, r/mHK-1 was found to cause a relaxation of the porcine coronary arteries [17] but to induce a contraction of the isolated rat urinary bladder [10], mouse and human uterus [18,19]. hHK-1 was also able to induce coronary vasodilatation followed with coronary vasorelaxation in the isolated guinea pig heart [20]. Numerous reports have focused on the involvement of the nonadrenergic noncholinergic system in the regulation of airway tone, demonstrating contractile properties for SP and NKA in human bronchi [21-25] and guinea pig airways [23,26]. Tachykinins released from the sensory unmyelinated C-fibers can cause the contraction of airway smooth muscle, an increase in vascular permeability, glandular secretion, and in cholinergic neurotransmission [27]. Tachykinins have been also involved in the recruitment and the activation of inflammatory cells such as mast cells [28], eosinophils [29], neutrophils [30,31], lymphocytes [32], monocytes and macrophages [33]. Tachykinins are also produced by immune and inflammatory cells airway smooth muscle cells, endothelial and epithelial cells, and fibroblasts [3,14,34-36]. This non neuronal production may be involved in the pulmonary effects of tachykinins. These peptides can induce bronchoconstriction in man, asthmatics being more sensitive than normal subjects, in agreement with the *in vitro *enhanced sensitivity and maximal response to tachykinins of human bronchi pretreated with serum from patients with atopic asthma [37]. However, in contrast to the characterization of SP-or NKA-mediated effects, little is known about the expression of hHK-1 and the contractile and inflammatory effects of this peptide in human airways. Thus, the aims of the present study were to determine the presence of tachykinins, tachykinin receptors and tachykinins degrading enzyme neutral endopeptidase (NEP) mRNAs, and hHK-1 protein in human bronchial tissues, and to characterize the effects of hHK-1, EKA/B (common C-terminal decapeptide of EKA and EKB [6,38]), EKC and EKD in human and guinea-pig isolated airways. Finally, effects of hHK-1 on the production of cytokines by explants of human bronchi or lung parenchyma and by human lung macrophages were assessed in comparison to those of SP. We report for the first time the endogenous expression of *TAC4 *and hHK-1 in human bronchi, together with a role of hHK-1 and endokinins in the contraction of human and guinea pig airways.

## Methods

### Human bronchi and guinea pig airways preparations

Human bronchial tissues were removed from 47 patients undergoing surgical resection at Foch Hospital (Suresnes, France) or Val d'or Clinic (Saint Cloud, France) for lung cancer (31 men and 16 women; age = 64 ± 9 years). Just after resection, segments of human bronchi with an inner diameter (ID) of 1 to 3 mm were taken as far as possible from the malignant lesion. Male Hartley guinea pigs (Charles River, L'Arbresle, France) weighing 300 to 350 g were sacrificed by cervical dislocation, and tracheas and proximal bronchi were removed. After the removal of adhering lung parenchyma and connective tissues, rings from human bronchi (5-7 mm long, 0.5-1 mm ID) and guinea pig trachea (3 mm long, 3 mm ID) or proximal airways (3 mm long, 1 mm ID) were prepared. 8 to 24 segments of human bronchi were obtained from each patient, whereas 8 trachea segments and 2 to 3 main bronchi segments were obtained from each guinea pig. For RT-PCR analysis, human bronchi were isolated within 1 hour after resection, immediately disrupted and homogenized in TRIzol reagent (Invitrogen) with a Potter Elvehjem homogenizer, and homogenates were kept frozen at -80°C until mRNA extraction. Experiments with human lung tissues were approved by the Regional Ethics Committee for Biomedical Research and animals were used as recommended by animal care guidelines.

### Reverse Transcriptase-Polymerase Chain Reaction (RT-PCR)

Total RNA was extracted from human bronchi (*n *= 4) using TRIzol reagent. After a DNase step (DNase I, Invitrogen), total RNA (1 μg) was reverse-transcribed using a High Capacity RNA-to-cDNA Synthesis Kit (Applied Biosystems, Les Ulis, France). The resulting product (cDNA) was used as template in endpoint or real-time PCR. Amplification was performed from 20 ng cDNA with Power SYBR Green PCR Master Mix (Applied Biosystems) in a MiniOpticon Real-Time PCR Detection System (Bio-Rad, Marnes-la-Coquette, France). Thermal cycling conditions were designed as follows: initial denaturation at 95°C for 10 min, followed by 40 cycles at 95°C for 15 sec and 60°C for 1 min. Total reaction volume was 25 μL with 300 nM of each reverse and forward primer. The primers used for tachykinins and their receptors were designed against sequences common to all described isoforms and were synthesized by Eurogentec (Angers, France). The primer pairs used for PCR were as follows: 5'-AAAGGGCTCCGGCAGTTC-3' and 5'-TGCAGAAGAAATAGGAGCCAATG-3' for *TAC1*; 5'-GAAGTCATGCATGTCACGTTTCTC-3' and 5'-GACTCTTCAAAAGCCACTCATCTCT-3' for *TAC3*; 5'-TACGGCGAAGCTGTGCATT-3' and 5'-TCACACAAGGCCCACACTGA-3' for *TAC4*; 5'-GTAGGGCAGGAGGAAGAAGATGT-3' and 5'-CAAGGTGGTCAAAATGATGATTGT-3' for *TACR1*; 5'-GAGGCCGATGACGCTGTAG-3' and 5'-CAAGACGCTCCTCCTGTACCA-3' for *TACR2*; 5'-ATATACCTGTCCACCGCAATGG-3' and 5'-CGCTTCCAGAACTTCTTTCCTATC-3' for *TACR3*. Expected amplicon sizes were 91, 110, 90, 85, 84 and 80 bp respectively. For *NEP*, primer pair was 5'-GGAGCTGGTCTCGGGAATG-3' and 5'-AGCCTCTCGGTCCTTGTCCT-3' [39] (amplicon expected size: 219 bp). To control for the recovery of intact cellular RNA and for the uniform efficiency of each reverse transcription reaction, a hypoxanthine phosphoribosyltransferase (HPRT) fragment was amplified by real-time RT-PCR (primer pair: 5'-TAATCCAGCAGGTCAGCAAAG-3' and 5'-CTGAGGATTTGGAAAGGGTGT-3'; expected size: 157 bp) on the same plate as that with tachykinins or tachykinins receptors cDNAs. The absence of secondary, non-specific amplification products in our experiments was assessed by analyzing melting curves and by separating PCR reaction products on agarose gel. The identity of each PCR product was established by DNA sequence analysis. With each sample, control samples without the RT step or with water instead of cDNA template were amplified to ensure there was no genomic DNA contamination and that all reagents were free of target sequence contamination. For each tachykinin and tachykinin receptor gene, a positive control sample of human fetal brain total mRNA (Ozyme, Saint Quentin en Yvelines, France) was also included in each run.

### In vitro bronchomotor responses

Human bronchial rings and guinea pig tracheal and bronchial rings were suspended on hooks in 5 mL organ bath containing a modified Krebs-Henseleit solution (NaCl 119, KCl 4.7, CaCl_2 _2.5, KH_2_PO_4 _1.2, NaHCO_3 _25 and glucose 11.7 mM), maintained at 37°C and oxygenated with 95% O_2 _and 5% CO_2_. An initial tension of 2 g was applied to tissues, according to previously described protocols [21,26]. Changes of tension were measured isometrically with Gould strain gauges (UF1; Piodem, Canterburry, Kent, UK); and were recorded and post-processed with IOX and Datanalyst softwares (Emka Technologies France, Paris). During the initial stabilization period (30 min), tissues were washed every 10 minutes with Krebs-Henseleit solution. Phosphoramidon was used to inhibit enzymatic degradation of tachykinins by NEP [21,40]. Phosphoramidon (10^-6 ^M) was added in organ bath with or without NK_1_-, NK_2_- or NK_3_-receptor antagonists (SR 140333, SR 48968 and SR 142801, 10^-7 ^M) after the first stabilization period. Antagonist concentrations were chosen based on their reported affinities for human tachykinin receptors [41-43] and on their ability to antagonize HK-1-induced responses at similar concentrations in other models [10,13,44]. Tissues were then equilibrated 1 hour and concentration-response curves to tachykinins and related peptides were established by applying cumulative concentrations of peptides at 5 to 10 min intervals in semi-logarithmic increments, or by applying a single concentration of peptide. Only one concentration-response curve to tachykinins was recorded in each strip, and each experiment was performed in duplicate. Maximal response was determined by a final addition of acetylcholine hydrochloride (ACh, 3 mM). Contractile responses to tachykinins and related compounds were expressed as percentage of that induced by ACh. The pD_2 _(defined as the negative log of the molar drug concentration that caused 50% of maximal effect) were calculated from the log concentration-effect curves. When the pD_2 _value was not assessable (maximal effect (E_max_) not reached), it was replaced by the -log EC_20 _(defined as the negative log of the drug concentration that caused 20% of maximal contraction with ACh). All values in the text and in the figures are expressed as arithmetic mean ± standard error of the mean (s.e.m) of duplicate experiments on tissues from the given (*n*) number of individuals or animals.

### Short-term culture of human bronchi and lung parenchyma explants and of lung macrophages

Explants of lung parenchyma and bronchi were prepared according to Mitsuta *et al. *[45]. Briefly, small bronchi (1 mm ID) removed from 4 patients and lung parenchyma from 6 patients were cut under sterile conditions into small fragments and rinsed once in RPMI 1640 supplemented with antibiotics (100 μg/mL streptomycin and 100 U/mL penicillin) and 2 mM L-glutamine. Explants were then conserved overnight at +4°C in RPMI supplemented medium. Fragments (≈ 50 mg) were pre-incubated in 12-well (bronchi) or 6-well (parenchyma) culture plates for 1 hour (37°C, 5% CO_2_) in the presence of phosphoramidon (10^-6 ^M) in 2.5 mL (bronchi) or 5 mL (parenchyma) of RPMI supplemented medium, before hHK-1 or SP (both 10^-9 ^to 10^-5 ^M) was applied.

Lung macrophages from 6 patients were isolated and cultured as previously described [46] and exposed to either hHK-1 or SP (both 10^-9 ^to 10^-5 ^M) after a 1-hour pre-incubation with phosphoramidon (10^-6 ^M). After a 24 hour incubation of bronchi and parenchyma explants or lung macrophages, supernatants were collected, centrifuged and frozen at -80°C until subsequent cytokine quantification.

### Cytokines and hHK-1 assays

Cytokines production (TNF-α, IL-6, IL-8, MIP-1α, MCP-1, ENA-78, GRO-α, MIG, and MIF) was assessed by measuring their concentrations in the culture supernatants with enzyme-linked immunosorbent assays (ELISA, Duoset Development System), according to the manufacturer's instructions (R&D Systems Europe, Lille, France). hHK-1 concentrations were determined with enzyme immunoassay (EIA) according to the manufacturer's instructions (Bachem, Weil am Rhein, Germany). Specifications of this EIA indicate absence of cross-reactivity with SP, NKA or NKB, and appropriate negative (RPMI alone) and positive (RPMI spiked with hHK-1) controls were included in the assay. Supernatants were diluted as appropriate and the optical density was determined at 450 nm with an MRX II microplate reader from Dynex Technologies (Saint-Cloud, France). Concentrations were expressed as pg per 100 mg tissue (bronchi and parenchyma explants) or pg per million cells (lung macrophages). The detection limits of these assays were 8 pg/ml for MIP-1α, 9 pg/ml for IL-6, 16 pg/ml for TNF-α, MCP-1 and ENA-78, 32 pg/ml for IL-8, GRO-α and MIF, and 62 pg/ml for MIG.

### Sources of chemicals and reagents

Substance P (RPKPQQFFGLM-NH_2_), [Sar^9^,Met(O_2_)^11^] substance P (selective for NK_1 _receptors), neurokinin A (HKTDSFVGLM-NH_2_), [β-Ala^8^]-NKA (4-10) (selective for NK_2 _receptors), neurokinin B (DMHDFFVGLM-NH_2_) were provided from Bachem and human hemokinin-1 (TGKASQFFGLM-NH_2_) from NeoMPS (Strasbourg, France). Custom synthesized endokinin A/B, endokinin C and endokinin D were supplied from Phoenix Pharma (Belmont, California, USA), SR 140333 ((S)1-(2-[3-(3,4-dichlorophenyl)-1-(3-isopropoxyphenylacetyl)piperidin-3-yl] ethyl)-4-phenyl-1-azoniabicyclo [2.2.2]octane chloride), SR 48968 ((S)-N-methyl-N-[4-acetylamino-4-phenylpiperidino-2-(3,4-dichlorophenyl) butyl]benzamide) and SR 142801 ((S)-(N)-(1-(3-(1-benzoyl-3-(3,4-dichlorophenyl)piperidin-3-yl)propyl)-4-phenylpiperidin-4-yl)-N-methylacetamide) were kindly provided by Dr Emonds-Alt (Sanofi Research Center, Montpellier, France) and dissolved in ethanol. Phosphoramidon (N-(α-L-rhamnopyranosyloxyhydroxyphosphinyl)-L-leucyl-L-tryptophan), penicillin/streptomycin stabilized solution, L-glutamine and acetylcholine hydrochloride were obtained from Sigma (Saint Louis, MO, United States); RPMI 1640 medium from Eurobio Biotechnology (Les Ulis, France). All tachykinins except NKB were dissolved in sterile distilled water and kept in aliquots at -20°C until used. Solutions of NKB were prepared in 20% dimethylsulfoxide and then diluted in distilled water. Maximal final concentrations of dimethylsulfoxide achieved in organ baths were found to have no effect on resting bronchial tone and on acetylcholine-induced responses.

### Statistical analysis of results

GraphPad Prism software (version 5.01 for Windows, GraphPad Software^®^, San Diego California, United States) was used to determine pD_2 _and E_max _and to perform a statistical analysis of the results, using ANOVA followed with Bonferroni post-tests. A *p *value lower than 0.05 (*p *< 0.05) was considered to be significant.

## Results

### Tachykinins, tachykinin receptors and neutral endopeptidase expression

**Figure 1:**
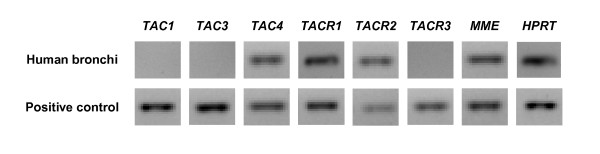
**Expression of tachykinin, tachykinin receptor and NEP mRNAs in human bronchi**. RT-PCR product of the housekeeping gene HPRT used as normalization standard is also represented. Equal aliquots of each cDNA sample (human bronchi or human fetal brain positive control) were amplified for 40 PCR cycles with their respective specific primer pairs. Since *TACR2 *was not expressed in human fetal brain, another bronchi sample was used as positive control for this gene.

In human bronchi, *TAC4*, *TACR1 *and *TACR2 *mRNAs were found in all samples whereas *TAC1 *and *TACR3 *mRNAs were not detected (fig. 1). A low *TAC3 *mRNA expression was found for one patient only, and *NEP *mRNA was expressed in high amounts in three of the four samples. All of these mRNAs were highly expressed in fetal brain positive control samples, except *TACR2 *mRNA which was not found in this tissue.

In addition to *TAC4 *mRNA expression, hHK-1 protein was found in the supernatants of bronchial explants (1.40 ± 0.31 pg/100 mg (*n *= 11)), parenchyma explants (1.15 ± 0.29 pg/100 mg (*n *= 11)) and lung macrophages (1.85 ± 0.89 pg/10^6 ^cells (*n *= 6)) cultured for 24 hours in the presence of phosphoramidon.

### Characterization of hHK-1- and endokinins-induced responses in human airways

#### Contractile effects of hHK-1 and endokinins in isolated human bronchi

**Figure 2:**
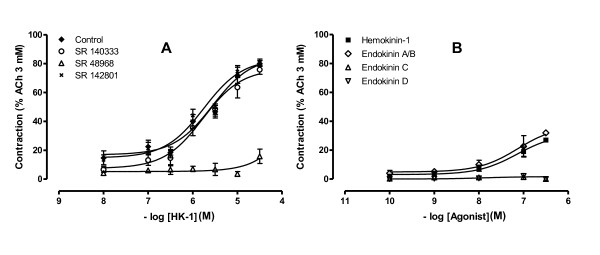
**(A) Cumulative concentration-response curves of hHK-1 on human bronchi (*n *= 5-12) in the absence (control) and presence of NK_1_, NK_2 _or NK_3 _receptor antagonists SR 140333, SR 48968 or SR 142801 (10^-7 ^M)**. (B) Cumulative concentration-response curves of hHK-1, EKA/B, EKC and EKD on human bronchi (*n *= 3). Experiments were performed in the presence of phosphoramidon (10^-6 ^M). Values are expressed in percentage (mean ± s.e.m.) of maximal contraction obtained with ACh 3 mM.

On human isolated bronchi and in the presence of phosphoramidon, hHK-1 produced concentration-dependent contractions reaching 80 ± 2% of the contraction induced by acetylcholine with a pD_2 _of 5.6 ± 0.2 (n = 12) (fig. 2A). In comparison, E_max _and pD_2 _values for the contractions induced by the NK_2 _receptor agonist NKA were 87 ± 1% and 8.5 ± 0.1 (curves not shown). EKA/B caused concentration-dependent contraction on human isolated bronchi and was equipotent to hHK-1 (respective -log EC_20 _of 7.2 ± 0.3 (*n *= 3) and 7.0 ± 0.5 (*n *= 3)), whereas EKC and EKD were devoid of any contractile activity (fig. 2B).

#### Effects of tachykinin receptor antagonists on cumulative additions of hHK-1 to human bronchi

The NK_2 _receptor antagonist SR 48968 (10^-7 ^M), completely abolished the contractile effects of cumulative additions of hHK-1 on human isolated bronchi, whereas the NK_1 _receptor antagonist SR 140333 (10^-7 ^M) only exerted a small but not statistically significant reduction of hHK-1-induced contraction at the lowest concentrations (10^-8 ^M-10^-7 ^M) (fig. 2A). Finally, the NK_3 _receptor antagonist SR 142801 (10^-7 ^M) did not alter the concentration-response curve to hHK-1.

#### Desensitization of the human tachykinin NK_1 _receptor

**Figure 3:**
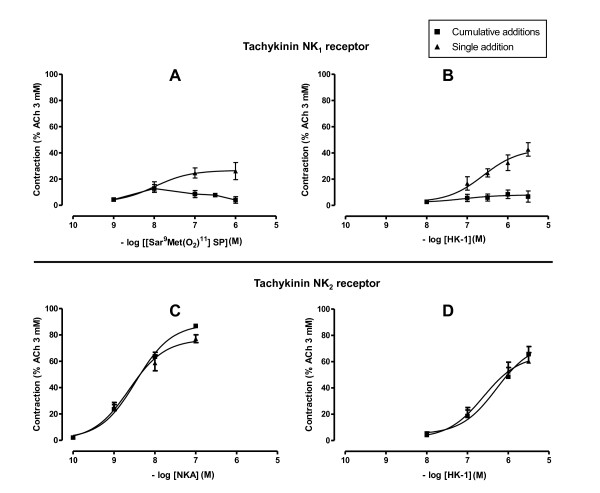
**Desensitization of tachykinin NK_1 _(A and B) and NK_2 _(C and D) receptors**. Human bronchi were pre-treated with SR 48968 (10^-7 ^M) (A and B) or SR 140333 (10^-7 ^M) (C and D) before cumulative or non cumulative additions of hHK-1 (B and D, *n *= 5), [Sar^9^,Met(O_2_)^11^] SP (A, *n *= 10) or NKA (C, *n *= 5). Experiments were performed in the presence of phosphoramidon (10^-6 ^M). Values are expressed in percentage (mean ± s.e.m.) of maximal contraction obtained with ACh 3 mM.

Since a rapid NK_1 _receptor desensitization has been reported in human isolated bronchi [22], and in order to clarify the role of the NK_1 _receptor in the responses to hHK-1, we compared the effects of single or cumulative additions of hHK-1 and of the specific NK_1 _receptor agonist [Sar^9^,Met(O_2_)^11^] SP. Experiments were performed in the presence of the NK_2 _receptor antagonist SR 48968 (10^-7 ^M) to block the NK_2 _receptor-mediated component. Cumulative additions of both peptides induced small contractions of human isolated bronchi (E_max _= 9 ± 3% and 13 ± 3%, respectively), characterized by inverted U-shaped concentration-response curves (fig. 3A and 3B). On the other hand, single additions of hHK-1 or [Sar^9^,Met(O_2_)^11^] SP did not lead to an inverted U-shaped curve but to a sigmoid response curve, and maximal contractions reached 43 ± 5% and 26 ± 7% respectively, with pD_2 _values of 6.6 ± 0.3 (*n *= 5-7) and 8.0 ± 0.4 (*n *= 10). In contrast, concentration-response curves for NKA and hHK-1 in the presence of the NK_1 _receptor antagonist SR 140333 (10^-7 ^M) were similar whatever the protocol used (fig. 3C and 3D).

#### Effects of tachykinin receptor antagonists on single addition of hHK-1 to human bronchi

**Figure 4:**
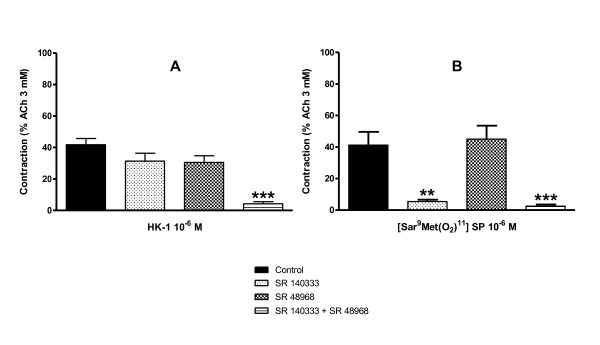
**Contraction induced with single additions of 10^-6 ^M hHK-1 (left graph, *n *= 6-12) or 10^-6 ^M specific NK_1 _receptor agonist [Sar^9^,Met(O_2_)^11^] SP (right graph, *n *= 6) on human bronchi in the absence (control) and presence of NK_1 _or NK_2 _receptor antagonists SR 140333 and SR 48968 (10^-7 ^M)**. Experiments were performed in the presence of phosphoramidon (10^-6 ^M). Values are expressed in percentage (mean ± s.e.m.) of maximal contraction obtained with ACh 3 mM. Statistical analysis was performed with one-way ANOVA followed with Bonferroni post-test. ** *p *< 0.01 and *** *p *< 0.001 *versus *paired control.

SR 140333 and SR 48968 reduced weakly but not significantly the response of human bronchi to a single addition of 10^-6 ^M hHK-1 (31 ± 5% and 31 ± 4% respectively, *versus *control 42 ± 4% (*n *= 6-12)) (fig. 4A). However, the association of both SR 140333 and SR 48968 was synergic and abolished the smooth muscle contraction. In contrast, the response to [Sar^9^,Met(O_2_)^11^] SP (10^-6 ^M), was specifically abolished by SR 140333 but unmodified by SR 48968 (fig. 4B).

#### Cross-desensitization of tachykinin NK_1 _receptor between hHK-1 and [Sar^9^,Met(O_2_)^11^] SP

**Figure 5:**
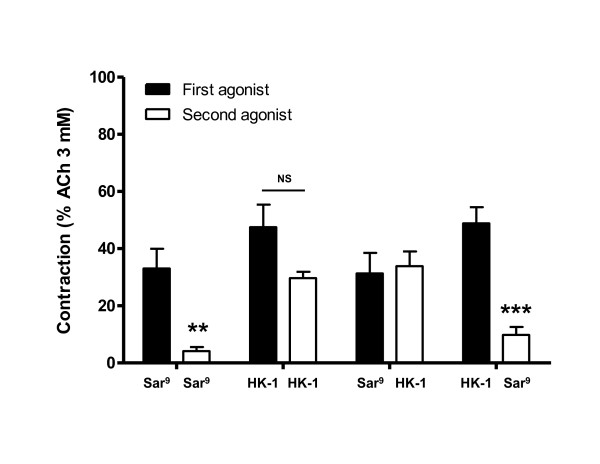
**Cross-desensitization of NK_1 _receptors after consecutive applications of hHK-1 (3.10^-7 ^M) and [Sar^9^,Met(O_2_)^11^] SP (10^-7 ^M) on human bronchi (*n *= 5)**. All combinations of hHK-1 and [Sar^9^,Met(O_2_)^11^] SP were assessed. Experiments were performed in the presence of phosphoramidon (10^-6 ^M). Values are expressed in percentage (mean ± s.e.m.) of maximal contraction obtained with ACh 3 mM. Statistical analysis was performed with two-way ANOVA for repeated measures followed with Bonferroni post-test. ** *p *< 0.01 and *** *p *< 0.001 for contraction obtained after the second application *versus *the first application. (Sar^9 ^= [Sar^9^,Met(O_2_)^11^] SP).

Since [Sar^9^,Met(O_2_)^11^] SP and hHK-1 are both able to induce a desensitization of NK_1 _receptors, we performed cross-desensitization experiments with the two compounds in order to assess if tissues desensitized with one peptide were still responsive to a subsequent addition of the other peptide. Fig. 5 shows that after an initial contraction induced by a single addition of [Sar^9^,Met(O_2_)^11^] SP (10^-7 ^M), the response to a second addition of this peptide was abolished (33 ± 7% for the first addition, 4 ± 1% for the second, *n *= 5, *p *< 0.01), whereas under similar conditions, after an initial addition of [Sar^9^,Met(O_2_)^11^] SP, the response to hHK-1 (3.10^-7 ^M) was maintained (32 ± 7% and 34 ± 5% respectively, *n *= 5). When hHK-1 was added in a first step to the bath, the response to [Sar^9^,Met(O_2_)^11^] SP was abolished, whereas the response to a second addition of hHK-1 itself was partially reduced (48 ± 8% and 30 ± 2% respectively, *n *= 5), suggesting a cross-desensitization between hHK-1 and [Sar^9^,Met(O_2_)^11^] SP for the NK_1 _receptor.

### Characterization of hHK-1- and endokinins-induced responses in guinea pig airways

#### Contractile effects of hHK-1 and endokinins in isolated guinea pig airways

**Figure 6:**
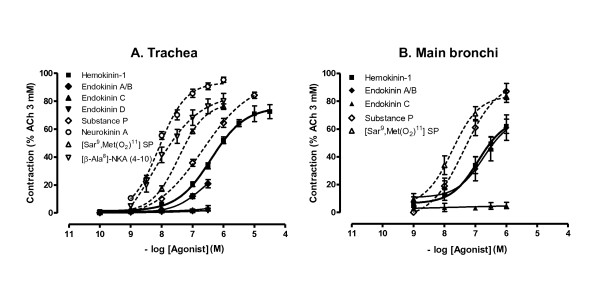
**Concentration-response curves to (A) cumulative additions of hHK-1, EKA/B, EKC, EKD, SP, NKA and specific NK_1 _([Sar^9^,Met(O_2_)^11^]SP) and NK_2 _([*β*-Ala^8^]-NKA (4-10)) receptor agonists on guinea pig trachea (*n *= 6-12); (B) cumulative additions of hHK-1, EKA/B, EKC, SP and [Sar^9^,Met(O_2_)^11^]SP on guinea pig main bronchi (*n *= 6-7)**. Experiments were performed in the presence of phosphoramidon (10^-6 ^M). Values are expressed in percentage (mean ± s.e.m.) of maximal contraction obtained with ACh 3 mM.

Human hemokinin-1 induced concentration-dependent contractions of the guinea-pig trachea (fig. 6A). This effect was reproducible and independent of the protocol used for the addition of hHK-1 (cumulative or noncumulative). Table 1 shows that hHK-1 potency was similar to that of SP, but was 11-fold lower than that of [Sar^9^,Met(O_2_)^11^] SP and 49- and 72-fold lower than that of the NK_2_-receptor agonists, NKA and [β-Ala^8^]-NKA (4-10), respectively. EKA/B (10^-8 ^M-10^-6 ^M) exerted similar effects to hHK-1, whereas EKC and EKD were without effect (fig. 6A). In guinea-pig isolated bronchi (fig. 6B), hHK-1 and EKA/B exerted similar effects but were less potent than SP and [Sar^9^,Met(O_2_)^11^] SP.

**Table 1:** Functional potencies and maximal effects of human hemokinin-1 and various tachykinin peptides on guinea-pig trachea.

Agonist	N	pD_2_	E_max _(% of Ach 3 mM)
hHK-1	12	6.4 ± 0.03	73 ± 5
EKA/B	6	ND	ND
SP	12	6.7 ± 0.1	84 ± 2
[Sar^9^,Met(O_2_)^11^] SP	12	7.5 ± 0.1	76 ± 2
NKA	6	8.1 ± 0.1	95 ± 2
[*β*-Ala^8^]-NKA (4-10)	6	8.3 ± 0.6	80 ± 5

hHK-1: human hemokinin-1, EKA/B: endokinin A/B, SP: substance P, NKA: neurokinin A

ND: not determined because an asymptote was not reached with the highest concentration (1 μM) applied.

Values are presented as pD_2 _and percentage of maximal contraction obtained with Ach 3 mM (mean ± s.e.m.) for *n *determinations.

#### Effects of tachykinin receptor antagonists on cumulative additions of hHK-1 to guinea pig airways

**Figure 7:**
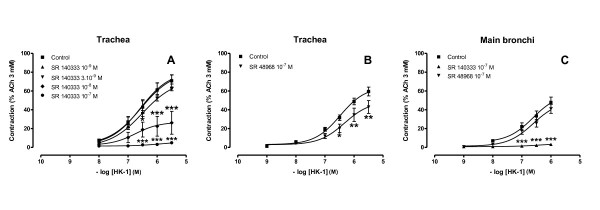
**Cumulative concentration-response curves to hHK-1 on guinea pig trachea (A and B) and main bronchi (C) pre-treated with NK_1 _or NK_2 _receptor antagonists**. (A) Cumulative additions of hHK-1 on guinea pig trachea in the absence (control) and presence of various concentrations of NK_1 _receptor antagonist SR 140333 (10^-9 ^to 10^-7 ^M) (*n *= 4-11). (B) Cumulative additions of hHK-1 on guinea pig trachea in the absence (control) and presence of NK_2 _receptor antagonist SR 48968 (10^-7 ^M) (*n *= 6). (C) Cumulative additions of hHK-1 on guinea pig main bronchi in the absence (control) and presence of NK_1 _or NK_2 _receptor antagonists SR 140333 and SR 48968 (10^-7 ^M) (*n *= 5-7). Experiments were performed in the presence of phosphoramidon (10^-6 ^M). Values are expressed in percentage (mean ± s.e.m.) of maximal contraction obtained with ACh 3 mM. Statistical analysis was performed with two-way ANOVA for repeated measures followed with Bonferroni post-test. * *p *< 0.05, ** *p *< 0.01 and *** *p *< 0.001 *versus *paired control.

Contractions induced by hHK-1 on the isolated guinea-pig trachea (fig. 7A and 7B) and main bronchi (fig. 7C) were abolished by the NK_1 _receptor antagonist SR 140333 (10^-7 ^M), and were altered to a lesser extent by the NK_2 _receptor antagonist SR 48968 (10^-7 ^M). In addition, fig. 7A shows that SR 140333 reduced maximal contractions induced by hHK-1 in the guinea-pig trachea, suggesting a non competitive antagonism in line with previous data on the rabbit pulmonary artery and on the guinea pig ileum [42].

### Effects of hHK-1 and SP on cytokine production by human bronchi or lung parenchyma explants and by lung macrophages

hHK-1 and SP up to 10^-5 ^M had no impact on TNF-α, IL-8 and MIP-1α production by bronchial explants (*n *= 4). Similarly, both peptides did not alter TNF-α, IL-6, MIP-1α, MCP-1, ENA-78, GRO-α, MIG, and MIF production by lung parenchyma (*n *= 6 different preparations) and TNF-α, IL-6, MIF, MIG and MIP-1α production by lung macrophages (*n *= 3 to 6 different preparations) (data not shown). LPS caused a clear-cut increase of these cytokines in all preparations.

## Discussion

In the present study we have demonstrated the expression of *TAC4 *transcript and protein in human bronchi and shown that hHK-1 and EKA/B exert a contractile effect in human and guinea pig airways. In human isolated bronchi, the response is mediated mainly through NK_2 _receptor stimulation, the NK_1 _receptor-mediated effect being unmasked in the presence of SR 48968 and subject to rapid desensitization. In guinea pig trachea and main bronchi, the response is mediated mainly through NK_1 _receptor stimulation and to a minor extent through NK_2 _receptors. The N-terminally extended form of human hHK-1, EKA/B, exerts similar effects to hHK-1 on both human bronchi and guinea pig airways, whereas EKC and EKD, peptides also derived from *TAC4*, did not induce functional responses. Finally, we have shown that hHK-1 did not alter cytokine production by human bronchi or parenchyma explants, or by human lung macrophages.

Our study shows that the *TAC4 *gene encoding for hHK-1 is constitutively present and expressed in human airways. Only a few numbers of studies have been devoted to the presence of *TAC4 *in human tissues, particularly in lung, and none of them has previously reported expression of hHK-1 protein. Indeed, *TAC4 *expression was not found in the mouse lung by northern blot analysis [3], but was demonstrated by semi-quantitative PCR in murine lung (mouse, gerbil) [4,5]. In human, a wide expression of *TAC4 *has been reported with a strong expression in tissues such as heart, skeletal muscle, skin, thyroid, spinal cord, placenta, adrenal gland, spermatozoa and blood circulating cells and a weaker expression in whole lung, kidney, testis and liver [4,6,39,47]. In contrast to *TAC4*, we have shown that *TAC1*, which encodes for SP and NKA, was not detected under our experimental conditions and that *TAC3 *was observed in only one of four samples. In a previous study, Pinto *et al. *showed in a human total mRNA master panel (BD Biosciences Clontech) that *TAC1 *and *TAC3 *mRNAs were undetectable in the lung, but they observed a low expression of these transcripts in samples of human bronchi obtained from patients who had undergone lobectomy or pneumectomy for lung carcinoma, a high expression being observed in pulmonary arteries [48]. Concerning the genes that encode for tachykinin receptors, we have identified the mRNA expression of *TACR1 *(NK_1 _receptor) and *TACR2 *(NK_2 _receptor), in agreement with Pinto *et al. *and in agreement with previous immunohistochemical evidences of NK_1 _and NK_2 _receptor expressions in human bronchial smooth muscle, bronchial glands and bronchial vessels [48,49]. We did not find *TACR3 *(NK_3 _receptor) expression whereas Pinto *et al. *identified this transcript in all assayed tissues [48]. These discrepancies in the results of tachykinin transcript expression could be related to differences either within human samples or to differences in expression patterns of tachykinin genes along the respiratory tract since we used smaller bronchi than in the work of Pinto *et al. *and since differences in the response to tachykinins have been reported according to the size of human bronchi [22]. It should be noted that we found *TAC4 *transcript and hHK-1 protein expressions in human bronchi similar in size (1 to 3 mm) to the bronchi used for the functional studies, substantiating a role for hHK-1 in the regulation of airway tone. Finally, our results showing NEP mRNA expression are also consistent with previous studies reporting a strong expression of NEP in human bronchi [50].

In human isolated bronchi pre-treated with phosphoramidon, hHK-1 exerts a contractile effect which was abolished by the NK_2 _receptor antagonist SR 48968, while the NK_1 _receptor antagonist SR 140333 only weakly reduced the effects of hHK-1 at low concentrations. In human bronchi, hHK-1 appears 800-fold less potent than NKA. This result is in agreement with previous data obtained on NK_2 _receptors either with CHO cells [4] or rabbit pulmonary artery [13].

A rapid functional desensitization of NK_1 _receptors has been reported with SP and specific NK_1 _receptor agonists in different tissues [51,52] including airways [22]. In addition, HK-1 has been reported to induce a desensitization of NK_1 _receptors in human embryonic kidney cells [11], rabbit jugular veins [13], U251 MG astrocytoma cells [53] and scratching behavior in rats [54]. We also observed desensitization of NK_1 _receptors in human bronchi, since the magnitude of the contractile response caused by the second application of the NK_1_-receptor specific agonist was lower than after the first addition, even with a 10-fold higher concentration. Such a desensitization can be due to receptor internalization, which is a common phenomenon for NK_1 _receptor signaling [55] and has already been described with hHK-1 on astrocytoma cells [53]. We have demonstrated a cross-desensitization between hHK-1 and [Sar^9^, Met (O_2_)^11^] SP substantiating NK_1_-receptor activation and desensitization by hHK-1. In addition, since a first exposure to [Sar^9^, Met (O_2_)^11^] SP was able to desensitize the NK_1 _receptor, preventing a second response to this specific NK_1_-receptor agonist, but was unable to prevent the response to hHK-1, these cross-desensitization experiments further substantiate the NK_2_-receptor mediated component of the contractile response to hHK-1. As expected in the single addition protocol, the contractile effect of [Sar^9^,Met(O_2_)^11^] SP was abolished by the NK_1 _receptor antagonist SR 140333 and unmodified by the NK_2 _receptor antagonist SR 48968. In contrast, the effect of hHK-1 was not inhibited by SR 140333 or SR 48968 when used alone, but was abolished by concomitant addition of the two antagonists, demonstrating that hHK-1 contracts bronchi through NK_1_- and NK_2 _receptors. However, it can also be suggested that [Sar^9^,Met(O_2_)^11^] SP and SP on the one hand, and hHK-1 on the other hand, may bind to different sites of the NK_1 _receptor and interact in a different manner with receptor antagonists [5,56].

In contrast with the results in human bronchi, SP and the specific NK_1_-receptor agonist produced maximal responses similar to those of NKA and hHK-1 in guinea pig airways providing evidence of the higher involvement of NK_1 _receptors in this animal species than in humans as already reported [23,40]. In support of this notion, hHK-1 exerted a contractile effect mainly through NK_1 _receptor stimulation since this effect was abolished by the NK_1 _receptor antagonist SR 140333, but was only weakly reduced in the presence of the NK_2 _receptor antagonist SR 48968. However, the NK_2 _receptors play a predominant role in guinea pig airways contraction since NKA is approximately 10-fold more potent than SP [23,40]. The weak effect of SR 48968 against hHK-1 induced bronchoconstriction in the guinea pig airways is likely explained by the higher affinity of hHK-1 for NK_1_- than for NK_2 _receptors in a preparation fully responsive to NK_1_-mediated response [4,10,13]. It is noteworthy that the potency of hHK-1 in the guinea pig airways was lower than that reported for r/mHK-1 in specific NK_1_-receptor animal tissues such as rabbit jugular vein [13], rat urinary bladder [10] and pig coronary artery [17].

In our study of cytokines production, we were not able to reproduce the weak TNF-α production that was observed in SP-stimulated human alveolar macrophages from healthy subjects [57]. This result may be related to the underlying disease or the smoking status of the patients that were all ex-smokers in our study since SP-induced TNF-α release is more pronounced in smokers [57]. SP-induced release of inflammatory mediators by human monocytes/macrophages still remains controversial and may have been related to the presence of endotoxin at low levels [58-61]. In addition to the lung macrophages, explants of lung parenchyma and bronchi did not produce pro-inflammatory cytokines in response to hHK-1 or SP, suggesting that hHK-1 may not be involved in lung inflammatory pathways through the release of these cytokines. However, hHK-1 may exert other inflammatory effects as already described for SP or NKA (reviewed in [36]).

SP expression has been reported in the human respiratory tract [62] and is increased in airways [63], bronchoalveolar or nasal lavages [64], sputum [65] or plasma [66] from asthmatics. It has been demonstrated that the antibodies used in such studies were directed against the C-terminal portion of SP, which is shared by hHK-1, leading to cross-reactivity with hHK-1 [5]. Immunoreactivity attributed to SP expression in human lungs may therefore be also related to hHK-1 expression. The use of specific assays for hHK-1 is required to evaluate the respective expression of SP and hHK-1 in the respiratory tracts of healthy subjects and in patients with asthma.

## Conclusion

In conclusion, our results provide evidence for a constitutive expression of *TAC4 *and hHK-1 in human bronchi. Our findings indicate that hHK-1 could induce contraction of human bronchi and guinea pig airways. This hHK-1-induced contraction could be mainly attributed to NK_2 _receptors in humans and to NK_1 _receptors in guinea pig. The absence cytokine release from lung explants and macrophages suggests that hHK-1 does not participate in airways inflammation by inducing the release of the pattern of cytokines measured in the present study. hHK-1 is therefore involved in the tachykinin-driven contractile response of human airways, but further studies are needed for a better understanding of hHK-1 involvement in airway diseases such as asthma.

## Competing interests

The authors declare that they have no competing interests.

## Authors' contributions

SGD carried out the molecular genetic studies, the contractile function studies, the cultures of lung explants, the immunoassays, participated to the interpretation of data, performed the statistical analysis and drafted the manuscript. EN participated to the contractile function studies and to the analysis and interpretation of data. AB and PAR participated to the cultures of lung explants ant to the immunoassays. ES provided human tissues and critically revised the manuscript. CA and PD conceived the study, participated in its design and coordination and drafted the manuscript. All authors read and approved the final manuscript.
